# Supplementary material for: The role of passion and affect in adolescents’ basketball participation: A self-determination theory perspective
Source: PLoS One. 2025 Oct 9;20(10):e0333503. doi: 10.1371/journal.pone.0333503 (PMC12510496; doi:10.1371/journal.pone.0333503)
Supplement: Attachment — Submitted filename: Review Report Passion and Affect.docx [file pone.0333503.s001.docx]

**Dear Editor and Authors,**

Thank you for the opportunity to review the manuscript titled **"The Role of Passion and Affect in Adolescents' Basketball Participation: A Self-Determination Theory Perspective."** The study addresses a relevant topic in sports psychology and offers valuable insights into motivational processes among youth athletes. However, several methodological, analytical, and conceptual issues should be addressed to enhance the rigor and clarity of the manuscript.

**Abstract (Page 8)**

The abstract briefly indicates the aim and main results of the study. It should also include effect sizes (e.g., β-values) alongside p-values to assist in describing the magnitude of the observed associations. The term "basketball engagement" must be clearly defined because it appears to be a composite score. An affirmation about the cultural context (China) and the cross-sectional nature would more appropriately situate the generalizability and limitations of the findings.

**Introduction (Pages 9-16)**

The introduction efficiently establishes the importance of basketball engagement and presents the main constructs of Self-Determination Theory (SDT) and the Dualistic Model of Passion (DMP). The relationship between types of passion (harmonious and obsessive) and affective states needs to be more strictly theorized, however. The review is based predominantly on Western studies; including more research from Eastern cultural contexts would offer greater theoretical grounding and transference. Hypotheses are clearly stated and conform to the theoretical line of thinking.

**Methods**

**Participants and Procedure (Page 16-18):**

Page 16: The sample is described as being from southern Chinese schools, but there is no explanation given for using this sampling. Generalizability of findings to other regions or to other cultures is doubtful.

Page 17: The gender distribution is seriously unbalanced (112 men, 286 women), which may undermine the validity of gender-based moderation analyses. This should be noted as a limitation.

Page 18: Referral to ethical approval and consent procedures is appropriate.

**Measures (Page 17-18):**

Page 17: Replacement of "academic activities" with "basketball" in the modification of the Passion Scale is reasonable, but there is no proof of the validity of this alteration in the new environment.

Page 18: The composite indicator of "basketball engagement" is constructed from two self-report items (days and duration). How these are reduced to one score (e.g., sum of z-scores) must be noted in detail. Validity and reliability of the composite measure have not been established.

**Analytical Approach (Page 19):**

Use of SEM is justified. Model fit indices are reported and within standard cut-offs, which is great.

But no mention of missing data treatment or analysis software/version (although Mplus is mentioned elsewhere).

**Results**

**Preliminary Analyses (Page 20-21):**

Page 20-21 (Table 2): Provides useful descriptive and correlational results. But the high negative correlation between gender and basketball participation (r = -.455, p < .001) describes the sample imbalance issue.

The use of MANOVA in order to compare group differences is appropriate, although post-hoc tests are not presented.

**Main Analyses (Page 22-27):**

Page 22: Structural model results are nicely presented. However, reporting only standardized coefficients (β) and no confidence intervals is limiting for interpretation.

Page 23: Gender and age moderation analysis is welcome, but the models' fit for these multi-group analyses is suboptimal (CFI/TLI ≈ 0.845). This needs to be stated as a limitation, and the results interpreted with caution.

Pages 23-24 & 27 (Table 5): The report states that gender differences were not significant in comparing coefficients (Table 5), but sometimes in results and discussion sections, the discussion appears to suggest gender differences. This must be synchronized so it would not create confusion.

**Discussion (Pages 28-33)**

Discussion is appropriately drawing inferences from results against SDT and DMP. Mediating role of affect is strongly highlighted.

Direct obsessive passion effect being non-significant is significant and is dealt with accordingly.

Page 30: However, weak and non-significant gender moderation effects need to be reported more categorically. The inference that "males and junior high students showed stronger pathways" appears overstated on the basis of statistical evidence.

Cultural context (e.g., collectivist values, academic pressure) is well integrated, though the analysis could further explore how precisely these help to generate passion and affect in Chinese adolescents.

Page 32-33: Practical implications are relevant but could be made more concrete. For example, what would "autonomy-supportive environments" look like in practice in Chinese education systems?

**Limitations (Page 33)**

The authors properly state several limitations, including regional sampling and cross-sectional design. Other limitations need to be stated:

The sample imbalance by gender and its potential impact on conclusions.

The use of a new, untested composite measure of basketball involvement.

The relatively poor fit of the multi-group SEM models.
