# Supplementary material for: The role of passion and affect in adolescents’ basketball participation: A self-determination theory perspective
Source: PLoS One. 2025 Oct 9;20(10):e0333503. doi: 10.1371/journal.pone.0333503 (PMC12510496; doi:10.1371/journal.pone.0333503)
Supplement: Attachment — Submitted filename: Response Letter to Reviewers Comments.docx [file pone.0333503.s002.docx]

Dear Editor and Reviewers,

We would like to express our sincere gratitude to you for your meticulous review and valuable insights on our manuscript titled “**The Role of Passion and Affect in Adolescents’ Basketball Participation: A Self-Determination Theory Perspective**” Your professional comments have been instrumental in enhancing the rigor, clarity, and completeness of our work, and we have carefully addressed each of your suggestions. Below is our detailed response to each comment, with references to the corresponding pages in the revised manuscript (note: page numbers refer to the updated version of our manuscript).

**1. Comments on the Abstract**

**Reviewer’s Comment**

The abstract briefly indicates the aim and main results of the study. It should also include effect sizes (e.g., β-values) alongside p-values to assist in describing the magnitude of the observed associations. The term “basketball engagement” must be clearly defined because it appears to be a composite score. An affirmation about the cultural context (China) and the cross-sectional nature would more appropriately situate the generalizability and limitations of the findings.

**Our Response**

We have revised the abstract to address all these points **(Abstract, Page 1)**:

Added key effect sizes (β-values) and confidence intervals for core pathways (e.g., “harmonious passion positively predicted basketball engagement (β = .280, 95% CI [.127, .432], p = .003) through enhanced positive affect; harmonious passion → positive affect: β = .495, 95% CI [.356, .635], p < .001; positive affect → basketball engagement: β = .253, 95% CI [.132, .373], p = .001”).

Clearly defined “basketball engagement” as “a composite score derived from adolescents’ self-reported number of days and duration of basketball participation (excluding school-arranged activities) over the past seven days, standardized via z-scores and modeled as a latent variable in SEM”.

Explicitly noted the cultural context (“data from 400 Chinese adolescents from southern China”) and cross-sectional design, and their implications for generalizability (“Given the cross-sectional design and sampling from southern China, the generalizability of our findings to other cultural contexts or regions of China should be interpreted with caution”).

**2. Comments on the Introduction**

**Reviewer’s Comment**

The introduction efficiently establishes the importance of basketball engagement and presents the main constructs of SDT and DMP. The relationship between types of passion (harmonious and obsessive) and affective states needs to be more strictly theorized, however. The review is based predominantly on Western studies; including more research from Eastern cultural contexts would offer greater theoretical grounding and transference.

**Our Response**

We have strengthened the theoretical framework and expanded the literature review to include Eastern context studies:

Incorporated studies from Chinese/Eastern contexts:“Sports, such as basketball, play an important role in China’s youth development programs [6]. The Chinese government actively promotes basketball in schools across the country, such as setting up basketball in physical education classes and after-school activities[5]...” (**Introduction, Page 4**)

Added a paragraph to clarify the mechanistic link between passion types and affect, grounded in SDT’s basic psychological needs: “Research based on DMP distinguishes two types of passion with different effects on sports engagement [20]...” (**Literature Review,** **Page 7-8**)

**3. Comments on Participants and Procedure (Methods Section)**

**Reviewer’s Comment**

The sample is described as being from southern Chinese schools, but there is no explanation given for using this sampling. Generalizability of findings to other regions or to other cultures is doubtful. The gender distribution is seriously unbalanced (112 men, 286 women), which may undermine the validity of gender-based moderation analyses. This should be noted as a limitation.

**Our Response**

We have supplemented the sampling rationale and acknowledged the gender imbalance in both the Methods and Limitations sections (**Page 29**):

Added an explanation for sampling from southern China: “The study’s sample was drawn exclusively from schools in southern China, which may limit the generalizability of findings to other regions with different cultural or educational contexts...”.

Explicitly noted the gender imbalance in the Methods section (“The sample has a gender imbalance, with 112 male and 286 female participants. This imbalance may slightly compromise the statistical power of gender-based moderation analyses...”).

**4. Comments on Measures (Methods Section)**

**Reviewer’s Comment**

Replacement of “academic activities” with “basketball” in the modification of the Passion Scale is reasonable, but there is no proof of the validity of this alteration in the new environment. The composite indicator of “basketball engagement” is constructed from two self-report items (days and duration). How these are reduced to one score (e.g., sum of z-scores) must be noted in detail. Validity and reliability of the composite measure have not been established.

**Our Response**

We have supplemented validation evidence for the modified scales and detailed the composite score construction:

For the modified Passion Scale: Added results of confirmatory factor analysis (CFA) to verify structural validity in the basketball context (**Page 12-13**).

For basketball engagement: To address the construction and psychometric properties of the “basketball engagement” measure, we have supplemented detailed information on score calculation, internal consistency (reliability), and criterion validity in the revised manuscript (**Page 13-14**).

**5. Comments on Analytical Approach (Methods Section)**

**Reviewer’s Comment**

Use of SEM is justified. Model fit indices are reported and within standard cut-offs, which is great. But no mention of missing data treatment or analysis software/version (although Mplus is mentioned elsewhere).

**Our Response**

We have added details on missing data treatment and analysis software :

For scale validation, Confirmatory Factor Analysis (CFA) and hypothesis testing of the structural model were performed in Mplus 8.3. Missing data were handled using Mplus’s default Full Information Maximum Likelihood method (FIML). (**Statistical Analysis, Page 15**).

**6. Comments on Preliminary Analyses (Results Section)**

**Reviewer’s Comment**

The use of MANOVA in order to compare group differences is appropriate, although post-hoc tests are not presented.

**Our Response**

Regarding the post-hoc tests, we note the SPSS warning that post-hoc comparisons (e.g., Tukey’s HSD) were not conducted because both the ‘gender’ and ‘school’ factors only have two groups each. In statistical practice, post-hoc tests (including Tukey’s HSD) are typically used when there are ≥3 groups to compare multiple pairwise differences (to control family-wise error rate). For factors with only 2 groups (e.g., male vs. female, Junior high school vs. Senior high school), a simple independent-samples t-test (or the corresponding contrast in ANOVA) is sufficient to test the binary group difference, as there is only one pairwise comparison to make.

**7. Comments on Main Analyses (Results Section)**

**Reviewer’s Comment**

Structural model results are nicely presented. However, reporting only standardized coefficients (β) and no confidence intervals is limiting for interpretation. Gender and age moderation analysis is welcome, but the models' fit for these multi-group analyses is suboptimal (CFI/TLI ≈ 0.845). This needs to be stated as a limitation, and the results interpreted with caution. The report states that gender differences were not significant in comparing coefficients (Table 5), but sometimes in results and discussion sections, the discussion appears to suggest gender differences. This must be synchronized so it would not create confusion.

**Our Response**

We have addressed these issues by supplementing confidence intervals, clarifying model fit limitations, and synchronizing gender difference.

Added 95% confidence intervals for all structural paths (e.g., “harmonious passion → positive affect: β = .495, 95% CI [.356, .635], *p* < .001; positive affect → basketball engagement: β = .253, 95% CI [.132, .373], *p* = .001”) (**Page 18**).

Explicitly noted the multi-group model fit limitation in both Results and Limitations sections:

Results: “While CFI/TLI values were slightly below the 0.900 threshold, RMSEA values met acceptable standards.” (**Page 19**).

Limitations: “In the multi - group analysis exploring the moderating effects of gender and age, the model fit was not ideal, with CFI/TLI around .840... (**Page 30**).

**8. Comments on Discussion**

**Reviewer’s Comment**

Weak and non-significant gender moderation effects need to be reported more categorically. The inference that “males and junior high students showed stronger pathways” appears overstated on the basis of statistical evidence. Cultural context (e.g., collectivist values, academic pressure) is well integrated, though the analysis could further explore how precisely these help to generate passion and affect in Chinese adolescents. Practical implications are relevant but could be made more concrete.

**Our Response**

We have revised the Discussion to address these points:

Removed the overstated inference in **Abstract** (“Additionally, no significant gender differences were found in this study. However, age differences emerged: junior high school students showed stronger pathways for both harmonious passion-basketball engagement and obsessive passion-basketball engagement...”).

Made practical implications more concrete for Chinese education systems: “(1) Implementing school-based programs that foster harmonious passion by emphasizing autonomy, enjoyment, and personal growth rather than performance pressure alone. For example, schools can design basketball curricula that offer students choices and set up student-led basketball clubs where students organize training sessions and matches by themselves...” (**Page 28**).

**9. Comments on Limitations**

**Reviewer’s Comment**

The authors properly state several limitations, including regional sampling and cross-sectional design. Other limitations need to be stated: The sample imbalance by gender and its potential impact on conclusions. The use of a new, untested composite measure of basketball involvement. The relatively poor fit of the multi-group SEM models.

**Our Response**

We have added these limitations to the “Limitations and Shortcomings” section (**Page 29**):

Sample gender imbalance: “(2) The sample had a gender imbalance (112 males, 286 females), which may have reduced statistical power to detect gender moderation effects...”

Composite measure of basketball engagement: “(6) The basketball engagement composite (days + duration) was newly adapted for this study. While we established internal consistency (α = .930) and criterion validity (correlation with harmonious passion, r = .272, p < .001), it has not been validated against objective measures (e.g., activity tracker data), which may introduce self-report bias.”

Multi-group SEM fit: “(4) In the multi - group analysis exploring the moderating effects of gender and age, the model fit was not ideal, with CFI/TLI around .840. This suggests the model might not fully capture the complex relationships...”

**Additional Correction**

During the revision process, we identified and corrected minor errors in the Sample sizes (**Abstract and Page 13**) and model fit of SEM (**Page 18**) that were overlooked in the initial submission. These corrections do not alter the main findings but improve the accuracy of data presentation.

Once again, we would like to thank you for your time, expertise, and constructive feedback. Your input has significantly improved the quality of our manuscript. We hope the revised version meets the journal’s standards, and we are happy to provide further clarification or make additional revisions if needed.

Sincerely,

Peng Tang

Hohai University
