# Supplementary material for: The role of passion and affect in adolescents’ basketball participation: A self-determination theory perspective
Source: PLoS One. 2025 Oct 9;20(10):e0333503. doi: 10.1371/journal.pone.0333503 (PMC12510496; doi:10.1371/journal.pone.0333503)
Supplement: Attachment — Submitted filename: Review Report Passion and Affect 1.docx [file pone.0333503.s003.docx]

**Dear Editor and Authors,**

Thank you for the opportunity to review the revised manuscript titled **“The Role of Passion and Affect in Adolescents' Basketball Participation: A Self-Determination Theory Perspective.”** The authors have done an exemplary job in addressing the previous round of comments. The revisions have significantly enhanced the manuscript's methodological rigor, clarity, and theoretical grounding. The study makes a valuable contribution to the literature on youth sports participation in a unique cultural context.

The responses to all previous concerns are satisfactory. The manuscript is now substantially stronger and is recommended for acceptance pending the following very minor final corrections:

**Abstract**: **Minor Point:** Please perform a final proofread to ensure flawless grammar and flow, particularly in the newly added sentences regarding the composite score and generalizability.

**Methods**: **Measures (Basketball Engagement):** The description of the composite score is now much improved. For ultimate clarity, please explicitly state the formula used to create the composite (e.g., "the mean of the standardized z-scores for days and duration") in the main text, even if it is implied.

**Results**: **Main Analyses:** The addition of confidence intervals for all structural paths is excellent and fully addresses the previous concern. No further action is needed here.

**Discussion**: **Page 30 (or relevant page):** The discussion of gender differences is now appropriately cautious and synchronized with the results. The removal of overstatements is noted and appreciated.

**Practical Implications:** The added concrete examples (e.g., student-led clubs, choice-based training) are perfect and effectively address how to implement autonomy-supportive environments in China.

**Limitations**: The new limitations regarding gender imbalance, the novel composite measure, and the multi-group model fit are now clearly stated and appropriately frame the interpretation of the findings.

In summary, the authors have been exceptionally responsive and thorough. The remaining points are minor and editorial in nature. Once these final small touches are complete, this manuscript will be ready for publication.

**Thank you for your diligent work on this revision.**
