# Supplementary material for: The role of passion and affect in adolescents’ basketball participation: A self-determination theory perspective
Source: PLoS One. 2025 Oct 9;20(10):e0333503. doi: 10.1371/journal.pone.0333503 (PMC12510496; doi:10.1371/journal.pone.0333503)
Supplement: Attachment — Submitted filename: Response_Letter_to_Reviewers_Comments_auresp_2.docx [file pone.0333503.s004.docx]

Dear Editor and Reviewer,

Thank you for giving us the opportunity to submit a revised version of our manuscript, “**The Role of Passion and Affect in Adolescents' Basketball Participation: A Self-Determination Theory Perspective**”. We are also deeply grateful to the editor and reviewer for exceptionally insightful and constructive comments, which have been invaluable in helping us to significantly improve the quality of our paper.

We have carefully considered all the feedback and have revised the manuscript accordingly. The changes made have strengthened the clarity, methodological rigor, and overall impact of our study. Below, we provide a point-by-point response to the final minor corrections suggested (note: page numbers refer to the updated version of our manuscript).

**1. Comments on Abstract**

**Reviewer’s Comment**

Regarding the Abstract: Please perform a final proofread to ensure flawless grammar and flow, particularly in the newly added sentences.

**Our Response**

We sincerely thank the reviewer for this suggestion. We have meticulously proofread the entire abstract, with special attention to grammar and sentence fluency. We have made several minor refinements to enhance readability and ensure the text meets a high standard of academic English **(Abstract, Page 1).**

2. **Comments on Methods**

**Reviewer’s Comment**

Comment 2: Regarding the Methods section (Measures - Basketball Engagement): For ultimate clarity, please explicitly state the formula used to create the composite score.

**Our Response**

We greatly appreciate this suggestion, which adds crucial clarity to our methodology. As recommended, we have now explicitly stated the formula used to create the composite score for basketball engagement. The revised text clearly indicates that the score was calculated as the product of the number of days and the average duration per session (Days × Duration), followed by standardization. We believe this revision leaves no ambiguity about how the variable was operationalized **(Page 13)**.

3. **Comments on Discussion**

We also note the reviewer’s positive feedback on our revisions concerning the results, discussion, practical implications, and limitations. We are pleased that these changes were found to be satisfactory.

Once again, we extend our deepest gratitude to the editor and the reviewer for their time, expertise, and diligent work throughout the review process. Their efforts have been instrumental in enhancing our manuscript. We hope that our revisions have adequately addressed all remaining points and that the manuscript is now deemed suitable for publication.

Sincerely,

Peng Tang

Hohai University
